# The Goat (*Capra hircus*) Mammary Gland Mitochondrial Proteome: A Study on the Effect of Weight Loss Using Blue-Native PAGE and Two-Dimensional Gel Electrophoresis

**DOI:** 10.1371/journal.pone.0151599

**Published:** 2016-03-31

**Authors:** Graziano Cugno, José R. Parreira, Enea Ferlizza, Lorenzo E. Hernández-Castellano, Mariana Carneiro, Jenny Renaut, Noemí Castro, Anastasio Arguello, Juan Capote, Alexandre M. O. Campos, André M. Almeida

**Affiliations:** 1 CIIMAR, Centro Interdisciplinar de Investigação Marinha e Ambiental, Universidade do Porto, Porto, Portugal; 2 Animal Science Department, Universidad de Las Palmas de Gran Canaria, Arucas, Gran Canaria, Spain; 3 IBET – Instituto de Biologia Experimental e Tecnologica, Oeiras, Portugal; 4 ITQB/UNL – Instituto de Tecnologia Química e Biológica, Universidade Nova de Lisboa, Oeiras, Portugal; 5 Department of Veterinary Medical Sciences, University of Bologna, Bologna, Italy; 6 Veterinary Physiology, Vetsuisse Faculty, University of Bern, Bern, Switzerland; 7 LIST – Luxemburg Institute of Science and Technology, Belvaux, Luxemburg; 8 Instituto Canario de Investigaciones Agrarias, Valle Guerra, Tenerife, Spain; 9 CIISA-Centro Interdisciplinar de Investigação em Sanidade Animal, Lisboa, Portugal; Biomedical Research Foundation, Academy of Athens, GREECE

## Abstract

Seasonal weight loss (SWL) is the most important limitation to animal production in the Tropical and Mediterranean regions, conditioning producer’s incomes and the nutritional status of rural communities. It is of importance to produce strategies to oppose adverse effects of SWL. Breeds that have evolved in harsh climates have acquired tolerance to SWL through selection. Most of the factors determining such ability are related to changes in biochemical pathways as affected by SWL. In this study, a gel based proteomics strategy (BN: Blue-Native Page and 2DE: Two-dimensional gel electrophoresis) was used to characterize the mitochondrial proteome of the secretory tissue of the goat mammary gland. In addition, we have conducted an investigation of the effects of weight loss in two goat breeds with different levels of adaptation to nutritional stress: Majorera (tolerant) and Palmera (susceptible). The study used Majorera and Palmera dairy goats, divided in 4 sets, 2 for each breed: underfed group fed on wheat straw (restricted diet, so their body weight would be 15–20% reduced by the end of experiment), and a control group fed with an energy-balanced diet. At the end of the experimental period (22 days), mammary gland biopsies were obtained for all experimental groups. The proteomic analysis of the mitochondria enabled the resolution of a total of 277 proteins, and 148 (53%) were identified by MALDI-TOF/TOF mass spectrometry. Some of the proteins were identified as subunits of the glutamate dehydrogenase complex and the respiratory complexes I, II, IV, V from mitochondria, as well as numerous other proteins with functions in: metabolism, development, localization, cellular organization and biogenesis, biological regulation, response to stimulus, among others, that were mapped in both BN and 2DE gels. The comparative proteomics analysis enabled the identification of several proteins: NADH-ubiquinone oxidoreductase 75 kDa subunit and lamin B1 mitochondrial (up-regulated in the Palmera breed), Guanine nucleotide-binding protein G(I)/G(S)/G(T) subunit beta-2 (up-regulated in the Majorera breed) and cytochrome b-c1 complex subunit 1, mitochondrial and Chain D, Bovine F1-C8 Sub-Complex Of Atp Synthase (down-regulated in the Majorera breed) as a consequence of weight loss.

## Introduction

Tropical and Mediterranean climates are characterized by the existence of two different seasons of varied duration, the dry and the rainy seasons. The rainy season is marked by abundant rainfall and consequently by pastures of adequate quantity and quality. The dry season, on the contrary, has a low or absent rainfall and consequently poor pastures. During the dry season, animals tend to lose weight in a phenomenon called Seasonal Weight Loss (SWL) that leads to significant decreases in productive and reproductive parameters and therefore in the income and food security of local populations. SWL is in fact considered to be one of the major drawbacks of animal production in the tropics as we have demonstrated in Western [1][2] and Southern Africa [3][4], as well as Western Australia [5][6] and the Canary Islands [7][8]. To counter SWL, farmers either use supplementation which is expensive and difficult to implement in remote regions or developing countries [9] or alternatively use breeds that over the course of domestication and selection have become naturally tolerant to SWL, such as the fat tailed sheep breeds in Eastern and Southern Africa [10].

The Canary Islands are an autonomous region of Spain, off the Atlantic coast of Northern Africa. The Canary Islands comprise seven islands with diverse and opposing micro-climates. Overall, the western region (La Palma, El Hierro, La Gomera and the Northern part of the island of Tenerife) are humid, but the eastern islands (Lanzarote, Fuerteventura, the Southern parts of Tenerife and Gran Canaria islands) are very dry [11]. These two groups of islands have different rain patterns that affect local ecosystems, agriculture, pasture abundance and animal production systems. Consequently, the Canary Islands are home to an unexpected diversity of animal breeds, descendants of ancestors imported from both Northern Africa and the Iberian Peninsula during the colonization of the islands. As a result, very different breeds were developed following the particular adaptations of the animals to the different climates of the islands. There are three dairy goat breeds: the Majorera, the Palmera and the Tinerfeña [12]. The Majorera goat, although presently found throughout most islands, is chiefly found in of Fuerteventura, Lanzarote and Gran Canaria [13]. The Palmera is found primarily on La Palma island, but also on others, particularly in Northern Tenerife [14]. Although Palmera and Majorera goats share a common ancestor, there are many differences. In fact, the Majorera goats are adapted to dry climates and therefore have an acquired resistance to SWL [15]. On the other hand, Palmera goats are adapted to rainy climates, and are susceptible to SWL [13].

Livestock selection aims at improving qualitative and quantitative efficiency. The study of alterations within the crucial metabolic agents during breed selection, will contribute to better understand the genomic and physiological relations leading to improved efficiency in animal production. SWL tolerance is at the forefront of such concerns within the context of breed improvement. Several studies have been previously reported, focusing primarily on the genome and on gene functions, nevertheless a growing number of the so-called post-genomics tools (Proteomics, Lipidomics, Metabolomics and Transcriptomics) have been playing an important role in understanding how biological systems work and interact. In particular, proteomics has been found to have a major role, not just in animal selection, but in diverse areas of animal and veterinary sciences [16][17] such as meat sciences [18][19], colostrum uptake [20][21][22] or the study of specific diseases such as chronic kidney disease in cats [23]. In fact, and although farm animal species poses some limitations [24], it is clear that significant results have been achieved in species such as cattle [25], rabbit [26] and pig [27].

The effect of SWL on physiological parameters in ruminants has long been the objective of this research team (e.g., lipid [28] and nitrogen [29] metabolisms), connected to productive characteristics [3]. Recent tendencies have focused on the effects of SWL on protein expression profiles, particularly through the comparison of breeds of domestic animals with different levels of adaptation to nutritional stress, as described for instance for the comparison of Wild Iberian and New Zealand White rabbits that led to the establishment of important difference between breeds at the level of structural proteins and glycolysis enzymes [30]. Such work was our first model for studies in larger production animals, in particular sheep and goats. The mammary gland is the most important organ in milk production. Contrary to the standard belief and from the morphologic point of view, the mammary gland is considerably different between sheep (*Ovis aries*) and goats (*Capra hircus*), changing also according considerably from breed to breed and lactation phase [31]. To understand the changes associated to SWL tolerance in dairy goats, it is important to conduct a proteomics-based study at the mammary gland level, specifically focusing on the secretory tissue and the mitochondria, the organelle responsible for cellular energy metabolism. In this work we used blue-native electrophoresis (BNE) and two-dimensional electrophoresis (2DE) coupled to mass spectrometry (MS) to elaborate the first dedicated 2DE and BNE mapping of the goat mammary gland secretory tissue. Such maps will be valuable information to researchers working on the mitochondrial proteomics of the goat mammary gland. Furthermore 2DE was performed to understand the changes in the mitochondrial proteome in the mammary gland of two breeds of goats with different levels of adaptation to nutritional stress: the Majorera (tolerant) and the Palmera (susceptible).

## Material and Methods

### Location, animals and nutritional treatments

As previously described [7][31], the study was conducted at the experimental farm of the Faculty of Veterinary Medicine of the ULPGC–*Universidad de Las Palmas de Gran* Canaria (Arucas, Gran Canaria, Spain) with 10 Majorera and 9 Palmera adult dairy goats. Husbandry details were as follows: goats had 3 lactations and had kidded in late February (serviced by natural mount using a buck of the same breed, five months prior to birth). Goats had a body condition score of 3 (in a scale of 5) which is the adequate body condition score for dairy goats during lactation. Animals were obtained from the experimental flock of the *Pico* Research Station (ICIA, Valle Guerra, Tenerife, Spain). Animals were classified as clinically healthy at the onset and through all the experimental period (22 days) and were admitted to the trial at mid-lactation (80 days on average). The goats were divided randomly into four sets, two for each breed: underfed and control groups. Animals were kept in a concrete floor shed with access to an external area with dirt soil limited by a 1.8 m fence. Following the practises of commercial farms in the Canary Islands and to minimize suffering and distress, animals were housed together in the same nutritional group, were free from rain and sunshine and had access to feed and water (under the nutritional trials guidelines) at a height ideal for their body size. Goats were milked, in a milking parlour, equipped with recording jars, at a vacuum pressure of 42 kPa, a pulsation ratio of 90 pulses/min, and a pulsation ratio of 60/40, in accordance with [32][33] following commercial practises in the area. At the beginning of the trial, live body weight (LBW) and daily milk yield were 45.5 kg and 1.60 L for Majorera Control (MC, n = 4), 50.6kg and 1.68 L for Majorera Underfed (MU, n = 5), 32.8 kg and 1.03 L for Palmera Control (PC, n = 6) and 40.6kg and 1.33 L for Palmera Underfed (PU, n = 4), as previously reported [7]. During the whole trial, all animals had free access to drinking water.

Aiming to reproduce field conditions in regions prone to drought and SWL where the pasture is characterized by low protein and high fibre content, the following nutritional restrictions were applied [3]. Animals from the underfed groups were fed on standard wheat straw and a vitamin-mineral supplement (underfed diet, in order to achieve a 15–20% reduction of their initial BLW by the end of the experimental period). The wheat straw basic composition corresponded to a low level of crude protein (approximately 30 g/kg dry matter), high amounts of fibre (420 g/kg dry matter) and low energy content (5.5 MJ/kg dry matter) [34]. In contrast, control animals were fed on a balanced diet, sufficient to cover their maintenance and lactation needs by using standard supplements found on the Canary Islands. As per [7] and [35], goats from the control groups were fed above-maintenance needs with maize, soy 44 (crude protein 44%), dehydrated lucerne, dehydrated beetroot, lucerne hay and a vitamin-mineral supplement. The control diet provided 1.81 kg of dry matter, 1.46 UFL, 133 g of metabolizable protein, 12 g of Ca and 6 g of P in accordance with the guidelines issued by the *Institut National de la Recherche Agronomique* (INRA). Goats from the underfed groups were fed with straw, representing 52% of the total UFL provided to the control group (1.81 kg of dry matter, 0.76 UFL, 41.13 g of metabolizable protein, 1.33 g of Ca and 0.66 g of P). Animals were fed daily at 07.00 am. The experimental period lasted 22 days from the point when the animals in the underfed groups had reached a stable decrease in relative LBW of 13–15%. At this point, LBW and daily milk yield were 48.2 kg and 1.99 L for MC and 44.1 kg with 0.22 L for MU, 33.9 kg and 1.15 L for PC and 35.4 kg and 0.17 L for PU, as previously reported [7]. Animals were monitored daily by the researchers involved in this study. Monitoring was done just before milking (09.00 am) and by the end of the day (18.00). Researchers monitored the animals for specific signs of possible health issues: correct stance, alert appearance and attitude, no prostration, no panting, no nervousness as well as usual food and water intakes.

### Sample collection

Mammary gland samples were collected by biopsy from the left half udder. Before biopsy collection, the udder was cleaned and disinfected using povidone-iodine. Local analgesia and anaesthesia were induced by intramuscular injection of Xylazine (Xilagesic 20%, Calier, Barcelona, Spain). A 2 mm incision was made to ease tissue collection and biopsy was taken using a scalpel blade. After sample collection, mammary gland was sutured using Safil 2/0 (Braun, Barcelona, Spain) and then sprayed with Terramicin (Terramicina spray, Pfizer, Madrid, Spain, containing oxytetracycline HCl and patent blue). Finally, prophylactic treatment was administered by intramuscular injection of Terramicin (Terramicina L.A., Pfizer, Madrid, Spain). Biopsies (1.5–2mg tissue) were then rinsed in sterile PBS, snap frozen in liquid Nitrogen and stored at -80°C until further analysis.

### Animal welfare disclaimer

All animal work herein described was conducted according to relevant international guidelines (European Union procedures on animal experimentation–Directive 2010/63/EU) that regulate the use of production animals in animal experimentation. This experiment was conducted with the approval of the Ethics Committee of the University of *Las Palmas de Gran Canaria* (1^st^ Process 2012) and the entire trial was conducted under the supervision of competent veterinary authorities. Author AM Almeida holds a FELASA (Federation of European Laboratory Animal Society Associations) grade C certificate that enables designing and carrying out animal experimentation under European Union regulations. Animals were subjected to conditions (housing, husbandry and feeding) very similar to those undertaken by local goat farmers. To avoid discomfort and distress, animals were provided with a shelter, appropriate feed troughs, and *ad libitum* access to water and were milked daily following the usual practises in dairy goat farming in the Canary Islands. Accordingly and during the trial, animals were monitored daily before milking and by the end of the day by the researchers involved in this study. Monitoring was made to detect any putative signs of discomfort or health related issues. These were absent. After the end of the trial, animals were monitored daily (twice a day: 08 am and 18.00 pm) for three weeks. Monitoring was conducted to prevent any complications at the biopsy site. None occurred. After these three weeks animals re-joined other animals in the experimental flock of the ULPGC veterinary faculty.

### Mitochondria extraction

Mammary gland biopsies (Majorera and Palmera breeds) were disrupted in liquid nitrogen with a tissue homogenizer (Biospec, Bartlesville, OK, USA) and homogenized in sucrose (0.35 M), EDTA (1.5 M) Tris (1 mM) and BSA (1%, w/v), pH 7.4 [36]. Additional homogenization was accomplished by sonication: 3 cycles at 60 Hz during 5s (VibraCell 50-sonics & Material Inc. Danbury, CT, USA). Mitochondrial fractions were subsequently obtained by differential centrifugation. The homogenates were first centrifuged for 10 min, at 700 *g* and 4°C to discard cell debris. A second centrifugation for 10 min at 8800 *g* was performed and the mitochondrial pellet (MP1) retained. MP1 samples were washed with aminocaproic acid (ACA) (750 mM), BisTris (50 mM), Na-EDTA (0.5 mM), pH 7.0 (BN sample buffer) [37], centrifuged and the resulting mitochondrial pellet (MP2) retained for proteomic analysis.

### Sample preparation and BN-PAGE/SDS-PAGE

MP2 samples from Majorera breed were homogenized in BN sample buffer to a final protein concentration of 1.4 mg/ml. Total protein was quantified using the Bradford method. Protein complexes were thereafter solubilized for 30 minutes at 4°C with the non-denaturing detergent n-Dodecyl-beta-D-maltoside (DDM) centrifuged (10000 *g*, 20 min.) and the supernatant retained for BN-PAGE (BN protein samples). The following protein:detergent (w/w) ratios were tested, 1:5, 1:7.5 and 1:10, to verify the influence of detergent concentration on protein solubilisation efficiency and the BN-PAGE protein profiles. Majorera breed protein samples were subsequently utilized to map the mitochondrial protein complexes by BN-PAGE. For this purpose BN protein samples were mixed with BN loading buffer (1/10 of sample volume) containing Coomassie blue G 250 (5%, w/v) and ACA (750 mM) solution. Coomassie blue dye binds to proteins and confer a negative charge for the separation by electrophoresis [37]. Mitochondrial protein complexes were thereafter separated by BN-PAGE using gradient gels (5% to 13%, w/v, acrylamide) as described previously [38]. The first dimension BN-PAGE lane was isolated, incubated in 2% SDS (w /v), 66 mm Na_2_CO_3_, 0.67% b-mercaptoethanol (b-ME, v / v) for 20 min to reduce proteins, and transferred immediately to the second dimension gel (SDS-PAGE) and protein subunits separated as described [38]. Protein bands (BN-PAGE) and spots (BN/SDS-PAGE) were visualized with colloidal Coomassie blue (CCB) [39]. Gel images were acquired with a GS-800 calibrated scanner (Bio-Rad, Hercules, CA, USA) and the protein patterns analysed with the Quantity-One or PDquest software’s (Bio-Rad, Hercules, CA, USA).

### Sample preparation and two-dimensional electrophoresis (2DE)

MP2 samples from Majorera and Palmera breeds were solubilized with urea (7M), thiourea (2M), CHAPS (4%, w/v), DTT (65 mM), ampholytes (0.8%, v/v) (2DE sample buffer; SB) for 1 hour at room temperature. The samples were centrifuged at 16000 *g* for 20 min and 20°C. The supernatants (2DE protein samples) were subsequently stored at -80°C before use to map the mitochondrial proteins with 2DE and for comparative protein expression analysis. The two-dimensional electrophoresis was based in the procedure previously described [40]. For each 2DE protein samples (400 μg of protein) were diluted to 300 μl in SB buffer. The protein samples were loaded in 17 cm, pH 3–10 IEF gel strips (Bio-Rad, Hercules, CA, USA) and proteins separated by isoelectric focusing (IEF) in a Protean IEF Cell (Bio-Rad, Hercules, CA, USA) with the following program: 16 h at 50V (strip rehydration); step 1, 15 min at 250 V; step 2, 3 h voltage gradient to 10,000V (linear ramp); step 3, 10,000V until achieving 60,000 V/h (linear ramp). After the first dimension proteins were reduced and alkylated with 10 mg/ml dithiothreitol and 25 mg/ml iodoacetamide as described [40]. Subsequently IEF gel strips were assembled on 12% (w/v) acrylamide SDS-PAGE slab gels (20cm×20cm×1 cm) and proteins separated by SDS-PAGE in a Protean II Xi Cell (Bio-Rad, Hercules, CA, USA) at 24mA per gel. Proteins were stained with CCB as previously described.

To evaluate differential protein expression among the four experimental groups (MC; MU; PC; PU), proteins were separated in 17 cm, pH 4–7 IEF gel strips and stained with fluorescent dye (Oriole, Bio-Rad, Hercules, CA, USA). 2DE Gel images were acquired with a Gel-Doc XR (Bio-Rad, Hercules, CA, USA). Protein spots were detected automatically with Samespots software (Progenesis, Newcastle upon Tyne, UK) as previously described [23]. Sensitivity parameters were reproduced for each gel image, and spot detection and matching were done manually. Protein spot intensities were normalized in terms of the total density in the gel image. For protein expression analysis, a master gel was obtained in the software, detecting all the spots present in the 2DE gel images. The presence or absence of spots and quantitative variations in spot intensities were analysed, comparing each spot’s intensity in each of all four experimental groups with each other. Only spots that were detected in at least two replicate gels were considered for expression analysis. Quantitative variations in spot intensity were statistically validated using t Student test (P < 0.05). Three 2DE gels (n = 3) were performed for each experimental condition, selected at random from the existing samples.

### Protein identification using LC-MS/MS

The BN-PAGE gel lane was divided into eight gel slices and subject to trypsin digestion. Peptide samples were next analysed on an Q Exactive mass spectrometer (Thermo Electron, Hemel Hempstead, UK) as described [41]. Samples were resolved on a 15 cm by 75μm inner diameter analytical column (New Objective, Woburn, MA, USA), which was packed in-house with Reprosil-Pur C18-AQ phase, 3 μm bead (Dr. Maisch, Germany). A 120 min gradient was used to separate the peptides. The Q Exactive mass spectrometer was operated in a “Top 10” data dependent acquisition mode. Precursor scans were performed at a resolving power of 60,000, from which 10 precursor ions were selected and fragmented. MS/MS peak lists were converted to mzXML format and searched using the MOWSE algorithm from Mascot version 2.3.01 (Matrix science) against UniProt_SwissProt database (541,762 sequences; 192,577,305 residues). Enzyme was set to trypsin allowing for up to 2 missed cleavages. Carbamidomethyl cysteine was set as a fixed modification and methionine oxidation as variable modification. Peptide mass tolerance was 20 ppm, and fragment mass tolerance 0.02 Da. Only hits with significant identity or extensive homology (p<0.05) were considered valid identifications. This work was conducted as an external service purchase to the Proteomics Facility, Sir William Dunn School of Pathology, University of Oxford, UK.

### Protein identification using MALDI-TOF/TOF

Protein spots were excised from 2DE and BN-PAGE/SDS-PAGE gels and proteins subjected to in-gel digestion using the protease trypsin [42]. The tryptic digests were desalted and concentrated using reversed phase micro-columns [43]. The peptides were eluted directly onto the MALDI plate using the matrix α-cyano-4-hydroxycinamic acid (5 mg/mL) prepared in acetonitrile (70%, v/v) and trifluoroacetic acid (0.1%, v/v). Protein identification was done by MALDI-TOF/TOF with an Applied Biosystem 5800 Proteomics Analyser (Applied Biosystems, Foster City, CA, USA) in MS and MS/MS mode. Spectra were externally calibrated using des-Arg-Bradykinin (904.468 Da), angiotensin 1 (1296.685 Da), Glu-Fibrinopeptide B (1570.677 Da), ACTH (1–17) (2093.087 Da), and ACTH (18–39) (2465.199) (Calibration Mix from 4700 Applied Biosystems). Ten s/n best precursors from each MS spectrum were selected for MS/MS analysis. The generated mass spectra were used to search the NCBI database restricted to Other Mammalia taxonomy (434586 sequences), with the algorithm MOWSE, from MASCOT server 2.3 (Matrix-Science). Two trypsin missed cleavages, carbamidomethylation of cysteine as fixed modification as well as four dynamic modifications (methionine and tryptophan oxidation, tryptophan dioxydation and tryptophan to kynurenin) were allowed. Mass accuracy was set to 100 ppm for parent ions and 0.5 Da for MS/MS fragments. Homology identification was retained with probability set at 95%.

## Results and Discussion

### BN/SDS-PAGE and characterization of mitochondrial protein complexes

**Fig 1:**
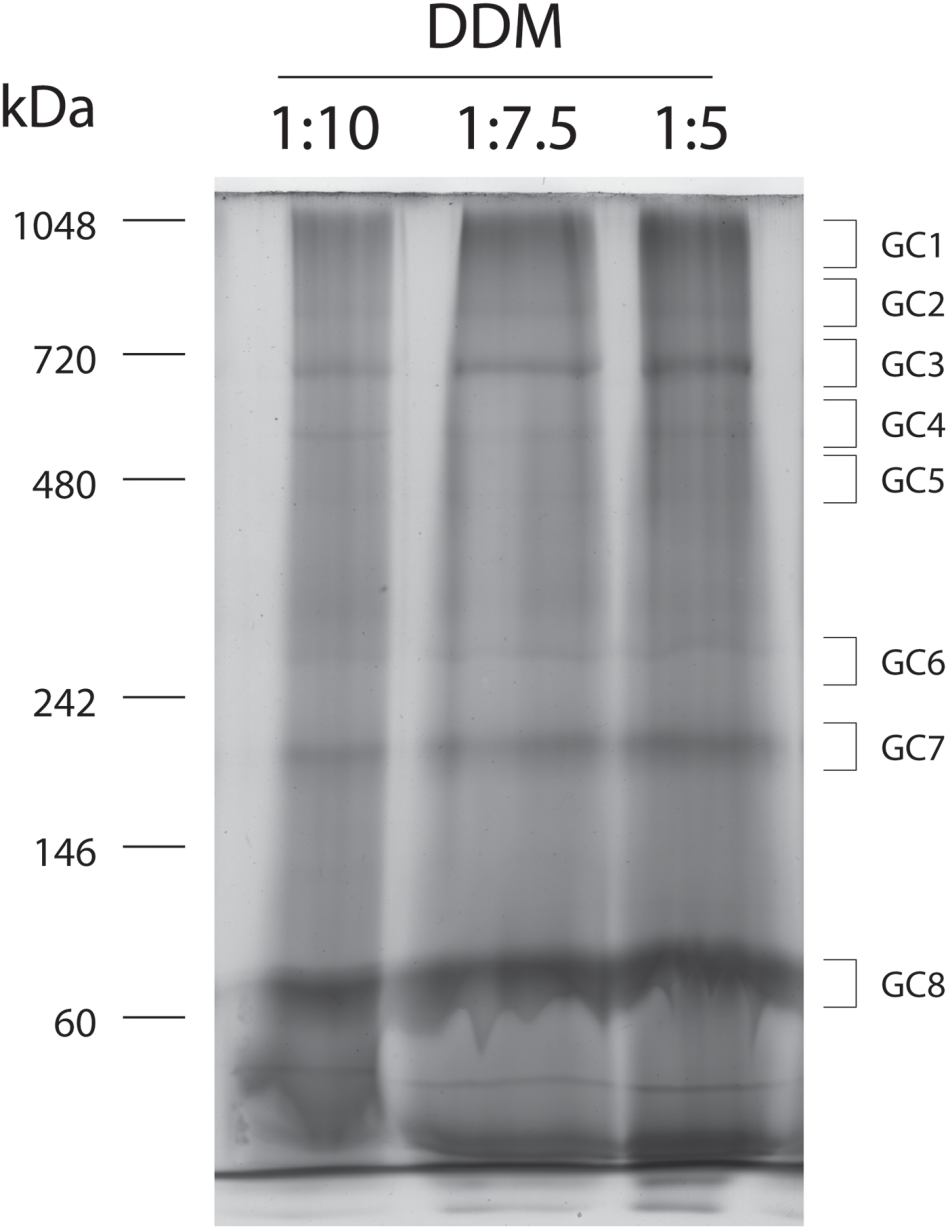
Separation of mitochondrial protein complexes from Majorera breed by BN-PAGE. Proteins were solubilized with the detergent DDM at a protein ratio of 1:10; 1:7.5 and 1:5 (protein: detergent, w/w). Gel stained with Coomassie Blue Colloidal. Gel bands were excised and analysed by nano-LC-MS/MS.

The solubilisation of the mitochondrial membrane protein complexes was attempted with the non-denaturant detergent DDM. Three protein:detergent (w/w) ratios (1:5, 1:7 and 1:10) were tested. The efficiency of the solubilisation was assessed by protein profiling in BN-PAGE gels (number of bands and intensity). Upon electrophoresis, similar profiles were reported for the three protein samples tested with, at least, eight protein bands (eight protein complexes) being resolved among samples (Fig 1). The molecular mass of the protein complexes in the BN-PAGE gels varied between 146 and 720 kDa or higher (Fig 1). The results indicate that DDM, within this concentration range, has no major influence in the solubilisation of membrane proteins. Moreover the results are consistent, for example, with the separation of mitochondrial membrane complexes I-V accomplished in different rat organs [44].

Protein complexes were subsequently excised from the gels and subjected to LC-MS/MS to gather a first insight on the protein complexes separated using this technique. The results of this analysis are resumed in Table 1. The LC-MS/MS analysis enabled the identification of several subunits of major protein complexes present in the mitochondria, the glutamate dehydrogenase complex and the respiratory NADH dehydrogenase (complex I), Succinate dehydrogenase, SQR (complex II), coenzyme Q: cytochrome c—oxidoreductase (complex III), cytochrome c oxidase, COX (complex IV) and ATP synthase, ATPase (complex V). Other subunits from cation transport ATPase, Voltage-dependent anion-selective channel, glutamate dehydrogenase, NAD(P) transhydrogenase, creatine kinase U-type complexes also were identified with the methodology (Table 1).

**Table 1:** Protein complexes, and respective subunits, identified by nano-LC-MS/MS. Detailed information of the identification of proteins is reported in S1 Table.

Protein band	Protein complex	Subunits	Identified subunits (gene identifier)
GC1	ATPase, **complex V**	**2**	ATP5A1, ATP5B
	Complex I	1	NDUFS1
	Complex II	1	SDHA
GC2	**complex I**	**7**	NDUFS1, NDUFV1, NDUFS2, NDUFS3, NDUFA13, NDUFB10, NDUFS8
	ATPase, complex V	3	ATP5A1, ATP5B, ATP5C1
	--	1	BDH1
	complex II	2	SDHA, UQCRC2
GC3	ATPase, **complex V**	**5**	ATP5A1, ATP5B, ATP5C1, ATP5F1, ATP5O
	**complex III**, cytochrome b-c1 complex	**3**	UQCRC1, UQCRC2, CYC1
	NAD(P) transhydrogenase	1	NNT
	COX complex, complex IV	1	MT-CO2
	Creatine kinase U-type	1	CKMT1
	--	1	BDH1
GC4	ATPase, complex V	2	ATP5A1, ATP5B
	glutamate dehydrogenase	1	GLUD1
GC5	glutamate dehydrogenase	1	GLUD1
	ATPase, **complex V**	**3**	ATP5B, ATP5A1, ATP5C1
	complex II	1	SDHA
	NAD(P) transhydrogenase	1	NNT
	--	2	Hspd1, BDH1
GC6	ATPase	1	ATP1A1
	COX complex, complex IV	2	COX4I1, COX6C
GC7	ATPase, complex V	2	ATP5B, ATP5A1
	NAD(P) transhydrogenase	1	NNT
	--	1	BDH1
G8	ATPase, complex V	2	ATP5B, ATP5A1
	Voltage-dependent anion-selective channel	3	VDAC1
	--		SLC25A6, SLC25A4

**Fig 2:**
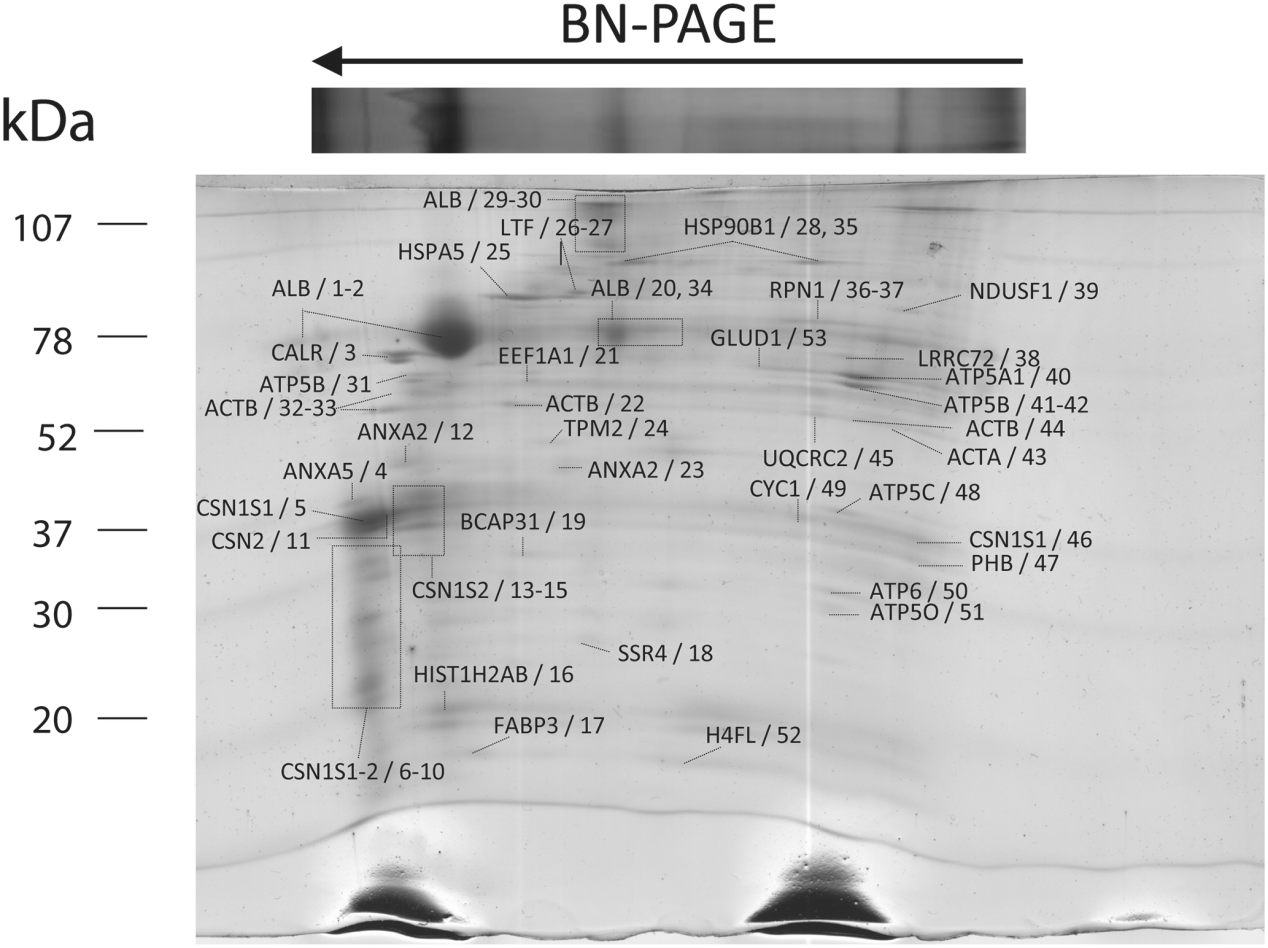
Second dimension separation of the subunits of mitochondrial protein complexes from Majorera breed, under denaturing conditions, BN-PAGE/SDS-PAGE. Identified proteins are referred in the figure by the gene identifier and reference number. Proteins were stained with CCB and identified with MALDI-TOF/TOF. Detailed information of protein identification is presented in S2 Table.

Following the first dimension (BN-PAGE), protein complexes can be denatured and the respective subunits separated in a second electrophoresis in denaturing conditions (SDS-PAGE). Coupled to MALDI-TOF mass spectrometry this method allowed to identify in total 53 proteins with molecular masses ranging between 20–117 kDa (Fig 2).

### 2DE of mitochondrial proteins

**Fig 3:**
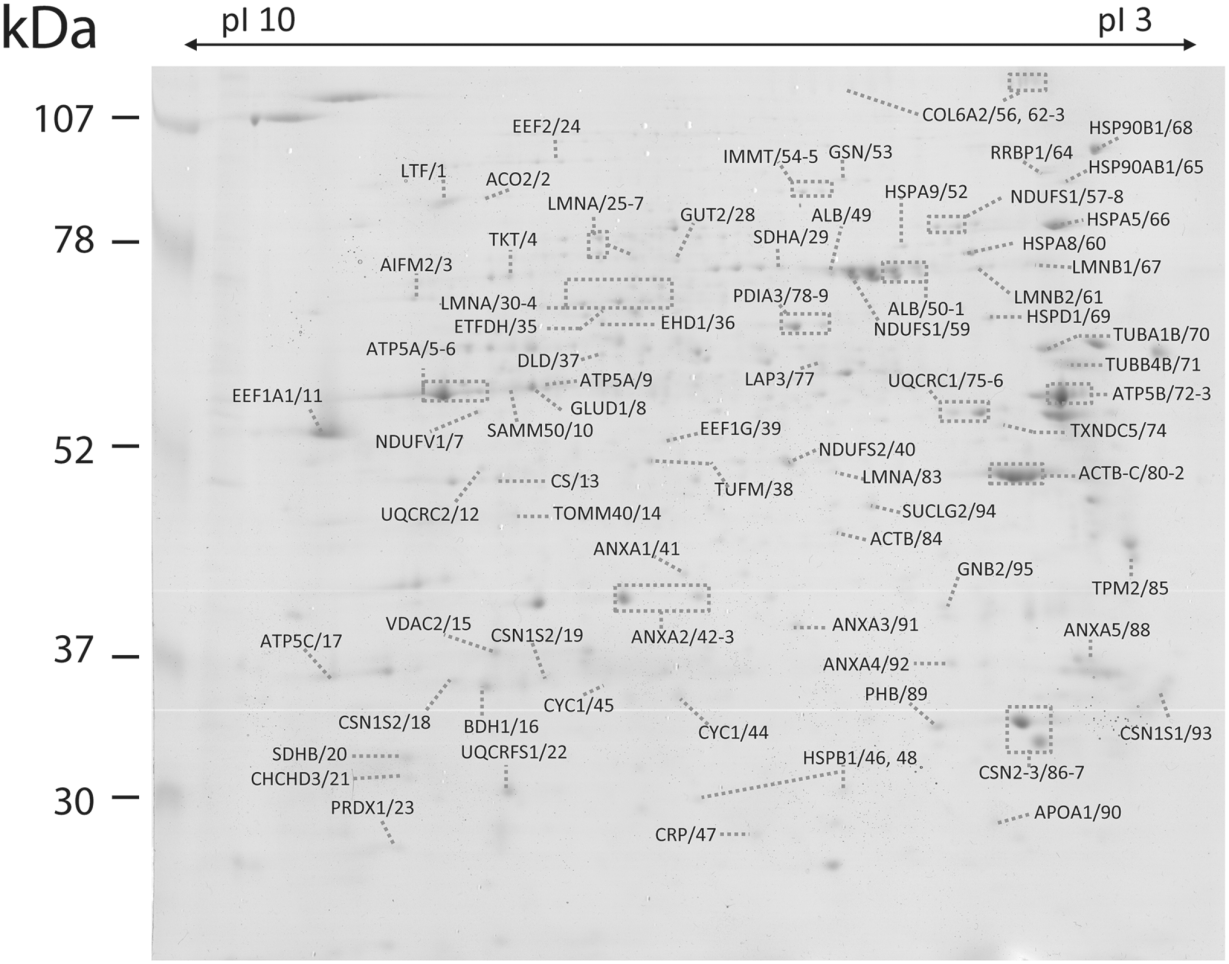
Two-dimensional gel electrophoresis of mitochondrial proteins from Majorera breed. Identified proteins are referred in the figure by the gene identifier and the reference number from the S1 Table. Proteins were separated within the pI and molecular mass intervals 3–10 and 20–117 kDa. Proteins were stained with CCB and identified with MALDI-TOF/TOF. Detailed information of protein identification is presented in S2 Table.

2DE was employed for a complementary mapping of the mitochondrial proteome (Fig 3). This conventional proteomic analysis method enables the information accomplished here to be reproduced and utilized in most of molecular and cell biology laboratories. Proteins with masses varying between 20 and 117 kDa were separated in large format gels along a pH interval of 3–10 (Fig 3). Ninety-five proteins were identified by MALDI-TOF/TOF, this higher number comparatively to BN/PAGE-SDS/PAGE could be attributed in part to the increased capability of 2DE to separate and resolve complex protein mixtures. Moreover in comparison to BN-PAGE the high resolution of 2DE has been exploited in the characterization of protein isoforms. In the present map several putative isoforms of ATP synthase subunits alpha and beta (ATP5A, ATP5B), prelamin (LMNA), protein disulfide-isomerase (PDIA), mitochondrial inner membrane protein (IMMT), NADH-ubiquinone oxidoreductase 75 kDa subunit (NDUSF) are reported (Fig 3, square delimited proteins).

### Gene ontology analysis and functional classification of proteins

**Fig 4:**
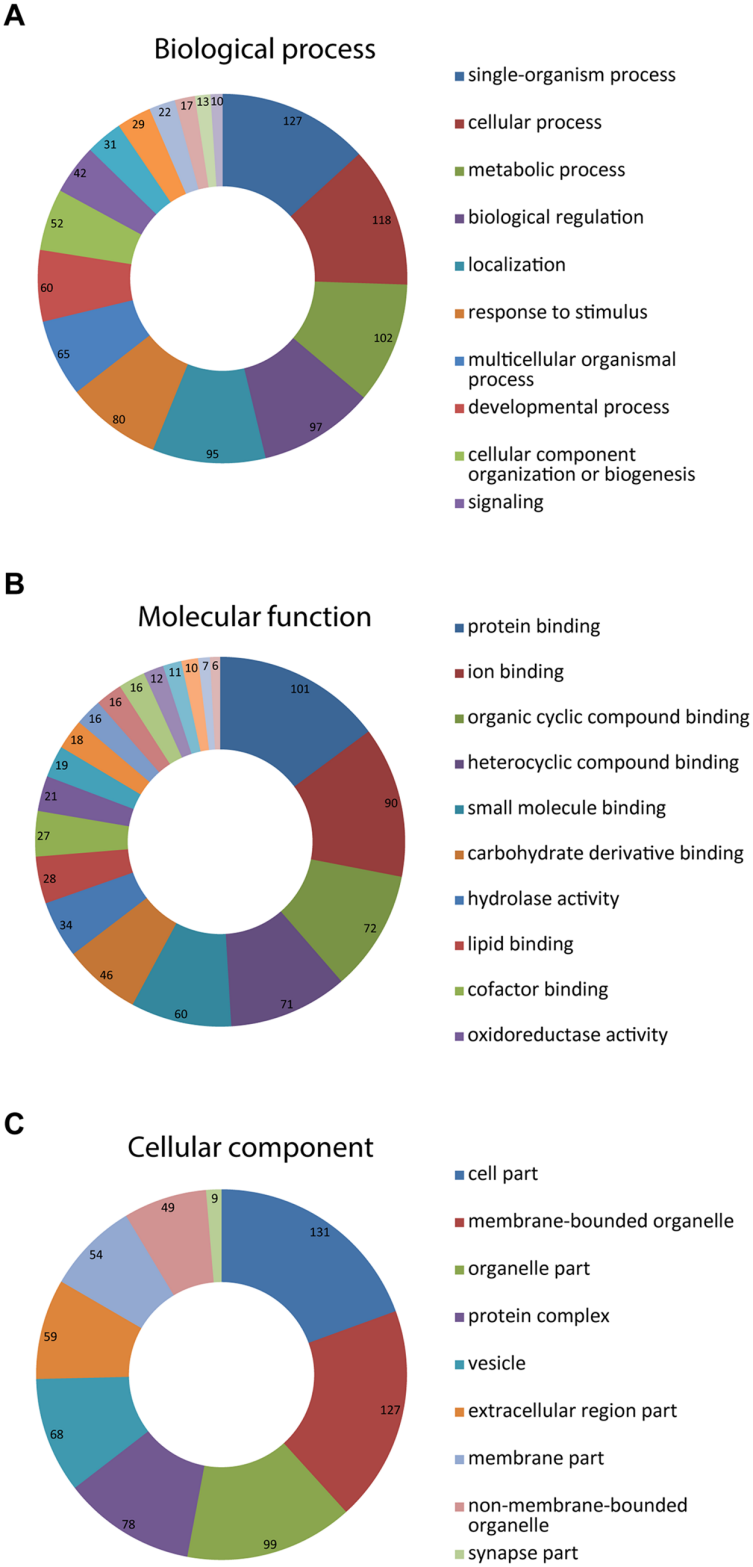
Protein distribution in the categories biological process (A—upper panel), molecular function (B—middle panel) and cellular component (C—lower panel), following analysis of GO term associations.

Functional classification of the proteins identified by MALDI-TOF/TOF was conducted in blast2go (https://www.blast2go.com/) tool and was based on gene ontology terms associated to each protein (Fig 4). The five most represented molecular activities displayed by the identified proteins are related to protein, ion, small molecule and organic cyclic and heterocyclic compound binding. Moreover the majority of these proteins seem to be involved in biological processes related with single organism, cell metabolism, biological regulation and response to stimulus (Fig 4). The majority of the proteins identified in 2DE and BN-PAGE have functions assigned directly to the mitochondria. Most of the proteins participate in citrate cycle (e.g. ACO2, CS, SDHB, SDHA, DLD, SUCLG2), oxidative phosphorylation and electron transport (ATP5A, NDUFV1, UQCRC2, ATP5C, UQCRFS1, ETFDH, NDUFS2, CYC1, NDUFS1, ATP5B, UQCRC1, ATP5O), amino acid metabolism (GLUD1), transport across the outer and inner membranes (TOMM40, CHCHD3, IMMT), signalling (VDAC2), lipid metabolism (BDH1, GUT2), protein folding (HSPA9, HSPD1, PHB), apoptosis (AIFM2), mRNA translation (TUFM). Mitochondria un-related proteins were also identified and are likely contaminants from other cellular structures and tissues, which could be attributed to an incomplete purification of the organelle. These include blood proteins (ALB), cytoskeletal (LMNA, GSN, LMNB2, LMNB1, TUBA1B, TUBB4B, ACTB, ACTC, TPM2), peroxisomal (PRDX1) and endoplasmic reticulum (RRBP1, RPN1) proteins and proteins involved in lipid metabolism (APOA1), amino acid metabolism (LAP3), transport (SAMM50, CSN1S2, CSN3, CSN1S1), endocytosis and exocytosis (ANXA1-5), mRNA translation (EEF1A1, EEF2, EEF1G), protein folding and processing (HSPB1, HSPA8, RRBP1, HSP90AB1, HSPA5, TXNDC5, PDIA3) and connection cell to cell (COL6A2).

### Differential protein expression

Differentially expressed proteins between the four experimental groups are presented in table 2. A total of 10 spots were found to have differential expression, allowing the identification of nine different proteins: NADH-ubiquinone oxidoreductase 75 kDa subunit, mitochondrial (2 spots), lamin B1, actin, cytoplasmic 2, casein beta, prelamin-A/C isoform 2, collagen alpha-1(VI) chain, Guanine nucleotide-binding protein G(I)/G(S)/G(T) subunit beta-2, cytochrome b-c1 complex subunit 1, mitochondrial, Chain D, Bovine F1-C8 Sub-Complex Of ATP Synthase.

**Table 2:** Protein spots showing differential abundance in Majorera and Palmera individuals subjected to food restriction. Values are reported as log normalized volumes. Statistical differences in comparison to control with P<0.05 (*).

Spot reference	Protein name	Majorera		Palmera		Accession Number	Matched Peptides^1^		Protein Score^2^
		Control	Underfed	Control	Underfed		MS	MS/ MS	
57	NADH-ubiquinone oxidoreductase 75 kDa subunit, mitochondrial	4.58±7.52e-002	4.5±0.47e-002	4,69±7.81e-002	4.5±6.91e-002*	gi|548454597	25	7	421
58	NADH-ubiquinone oxidoreductase 75 kDa subunit, mitochondrial	4.99±0.19	4.82±4.51e-002	5.04±1.47e-002	4.86±9.22e-002*	gi|548454597	29	6	529
62	prelamin-A/C isoform 2	4.91±0.28	5.47±0.43*	4.98±0.25	5.16±0.15	gi|472366893	16	5	288
63	collagen alpha-1(VI) chain	5.03±0.13	5.54±0.28*	5.06±0.21	5.26±017	gi|548452361	34	8	328
67	lamin B1	4.72±0.17	4.78±0.14	4.58±0.11	4.79±3.77e-002*	gi|296485609	38	7	359
73	Chain D, Bovine F1-C8 Sub-Complex Of Atp Synthase	5.44±3.94e-002	5.35±1.45e-002*	5.56±0.61	5.38±015	gi|306991567	22	9	817
75	cytochrome b-c1 complex subunit 1, mitochondrial	5.01±4.44e-002	4.86±9.01e-002*	5.05±9.16e-002	4.99±0.24	gi|548515658	21	7	679
80	actin, cytoplasmic 2	5.94±0.34	6.05±0.26	5.84±8.52e-002	5.95±5.25e-002*	gi|471367241	34	9	751
86	casein beta	4.81±7.52e-002	4.65±0.75	5.03±0.23	4.77±0.13*	gi|548470565	13	4	346
95	Guanine nucleotide-binding protein G(I)/G(S)/G(T) subunit beta-2	5.03±8.07e-002	5.21±9.36e-002*	4.98±0.16	5.0±0.13	gi|432101315	7	1	60

^1^Number of peptides matched and fragmented peptides in MALDI-TOF/TOF;

^2^Significant identification scores obtained with the Mowse algorithm (P<0.05)

NADH-ubiquinone oxidoreductase 75 kDa subunit is the core subunit of the mitochondrial membrane respiratory chain NADH dehydrogenase (Complex I), functioning essentially at the level of electron transfers from NADH to the respiratory chain (http://www.uniprot.org/uniprot/P15690). In our experiment, NADH-ubiquinone oxidoreductase 75 kDa subunit shows a pattern for decreased expression in the Palmera breed, with the pattern being consistent in both spots (57 and 58) identified as this protein. Ricci and co-workers [45] have demonstrated that during apoptosis and the production of reactive oxygen species (ROS) induced by stress, the p75 subunit of complex I of the electron transport chain is heavily cleaved by caspase, which may explain why did this protein expression decreased as a consequence of SWL. It is noteworthy to mention that such decrease was only significant in the SWL susceptible (Palmera breed) and not in the SWL tolerant (Majorera breed). Such results may be interpreted as a consequence of an exacerbated reaction to SWL-induced stress only on the Palmera mammary gland mitochondria, and not on the Majorera, finally indicating that the reduced expression of this protein may be considered as possible marker of tolerance to SWL in the mitochondria of the mammary gland.

The same expression pattern was also recorded for lamin B1. This protein has equally been found to be involved in the apoptotic process, being a component of the nuclear lamina and playing a critical role in maintaining nuclear architecture, regulating gene expression and modulating chromatin positioning [46]. According to these authors, lamin B1 protein over-expression has been associated to alterations in the lipid metabolism linked to age-dependent demyelination-suffering patients. Higher expression levels of lamin B1 in bovine mammary epithelial cells have also been linked to the late stages of lactation, particularly to mammary gland involution and associated apoptosis [47]. As higher expression levels for lamin B1 were found to be significantly different in the Palmera breed, it can be inferred that this breed is being significantly more affected by underfed than the Majorera animals that, despite the feed restriction levels, are still in an earlier phase of the mammary gland apoptosis and involution than Palmera goats. This protein may therefore also be considered as being related to SWL susceptibility.

Our results for spot 62 show that in the Majorera restricted group Prelamin A/C expression increases 10%, whereas in both Palmera groups, the results are very similar, albeit with a tendency to increase (3%) in the restricted group. This protein expression has been associated to cell senescence [48] corroborating our results. Contrariwise, it has also been demonstrated that albeit the protein is down-regulated in the interstitial fluid of leiomyoma patients [49], it is also up-regulated in malignant pleural mesothelioma patients [50], suggesting for prelamin A/C a still unclear and undefined role in the context of cellular growth or senescence. A similar profile was also detected for collagen alpha-1(VI) chain in our study. The protein has been found to be up-regulated in different tissues, being considered a marker of diverse types of cancer [51][52][53], and also a biomarker of gestational diabetes in omental adipose tissue [54]. All these conditions are characterized by intense cell proliferation and morphogenesis, clearly the opposite situation of both restricted groups in our trial. It seems therefore that further studies are necessary to understand the role of this protein in the goat mammary gland.

Guanine nucleotide-binding protein subunit beta (spot 95) has long been described as a protein involved in signalling systems in different types of mammalian cells, being particularly important in mechanisms such as cell growth [55]. Data from our study point out to an up-regulation of this protein in the mammary gland of the tolerant breed (Majorera) as a consequence of weight loss, whereas no changes were observed for the susceptible breed (Palmera). Such results indicate that in the Majorera breed, and despite the weight loss and the associated reserve mobilization, signalling systems leading to cell growth (and therefore mammary gland function) are still occurring and indeed increasing, whereas in the Palmera breed, cell growth seems to have reached a standstill. Such results indicate that this protein could be considered as an interesting indicator of SWL tolerance in the goat mammary gland.

Spot 75 was identified as cytochrome b-c1 complex subunit 1. This is a mitochondrial protein involved in oxidation/reduction process and playing a major role in the cell’s electron transport and respiratory chain. It has long been described as a marker for ageing in the skeletal muscle female humans [56]. More recently, this protein has also been described as playing a significant role for instance in ovine cellular (Corpus Luteum) regression [57]. In our experiment, the expression of the protein is maintained in the susceptible breed, but decreasing in the tolerant breed. This could be interpreted as a better adaptation to SWL by the Majorera breed in a response to diminish mammary gland regression by reducing the expression of cytochrome b-c1 complex subunit 1. On the contrary, Palmera animals are still maintaining the same expression levels of this protein lacking therefore this important mechanism of adaptation leading to the preservation of mammary gland function and lactation.

Spot 73 (Chain D, Bovine F1-C8 Sub-Complex of ATP Synthase) is another enzyme involved in energy metabolism [58]. The expression of the protein decreased similarly (2% decrease) in both breeds as a consequence of SWL, albeit significant differences were only recorded for Majorera goats. This is an expectable result as SWL leads to energy production reduction in the cell. The similar decreases for both groups indicate the importance of this protein as a putative mammary gland biomarker of SWL, but not of SWL tolerance.

## Conclusions

In this study, we conducted for the first time an exhaustive study of the goat mammary gland mitochondrial proteome. We have thoroughly mapped the proteins in both their Native organization and under denaturing conditions, respectively using BN-PAGE and standard 2DE. Furthermore and as this is, to the best of our knowledge, the first application of the BN-PAGE technique to mammary gland mitochondria, we have also contributed to the establishment of adequate and reproducible laboratorial methodology of use in this type of tissue. We have finally studied the protein expression changes in the mammary gland of two goat breeds with different levels of tolerance to SWL, highlighting several proteins that showed differential expression between breeds and nutritional treatments and as such, and upon adequate validation, could be proposed as putative biomarkers of tolerance to SWL and maintenance of lactation and mammary gland function in these animals. Results indicate that SWL tolerance and milk production under SWL are related to proteins involved in the apoptotic and cell senescence processes, with higher expression results in the Palmera breed, whereas the Majorera breed maintains the expression of these proteins at constant level. The control of apoptosis seems therefore to be the key link to understand SWL tolerance and its links to the production performances of these animals. These results furthermore serve as a basis for studies in other domestic dairy ruminants, particularly sheep and cattle. Understanding the molecular basis of tolerance to SWL is a complex task. It would therefore be interesting to conduct further research at the transcriptomics, proteomics (extra mitochondrial), lipidomics and metabolomics levels.

## Supporting Information

S1 Table Protein identification by nano-LC-MS/MS analysis (XLSX)

S2 Table Protein identification by MALDI-TOF/TOF analysis. (XLSX)
